# PFAS Biotransformation Pathways: A Species Comparison Study

**DOI:** 10.3390/toxics11010074

**Published:** 2023-01-12

**Authors:** Richard C. Kolanczyk, Megan R. Saley, Jose A. Serrano, Sara M. Daley, Mark A. Tapper

**Affiliations:** 1 Great Lakes Toxicology and Ecology Division, Center for Computational Toxicology and Exposure, Office of Research and Development, US Environmental Protection Agency, 6201 Congdon Blvd., Duluth, MN 55804, USA; 2 Oak Ridge Institute for Science and Education, Great Lakes Toxicology and Ecology Division, 6201 Congdon Blvd., Duluth, MN 55804, USA

**Keywords:** PFAS, biotransformation, metabolism, fish, biodegradation, pathway, MetaPath, comparison

## Abstract

Limited availability of fish metabolic pathways for PFAS may lead to risk assessments with inherent uncertainties based only upon the parent chemical or the assumption that the biodegradation or mammalian metabolism map data will serve as an adequate surrogate. A rapid and transparent process, utilizing a recently created database of systematically collected information for fish, mammals, poultry, plant, earthworm, sediment, sludge, bacteria, and fungus using data evaluation tools in the previously described metabolism pathway software system MetaPath, is presented. The fish metabolism maps for 10 PFAS, heptadecafluorooctyl(tridecafluorohexyl)phosphinic acid (C6/C8 PFPiA), bis(perfluorooctyl)phosphinic acid (C8/C8 PFPiA), 2-[(6-chloro-1,1,2,2,3,3,4,4,5,5,6,6-dodecafluorohexyl)oxy]-1,1,2,2-tetrafluoroethanesulfonic acid (6:2 Cl-PFESA), *N*-Ethylperfluorooctane-1-sulfonamide (Sulfuramid; N-EtFOSA), *N*-Ethyl Perfluorooctane Sulfonamido Ethanol phosphate diester (SAmPAP), Perfluorooctanesulfonamide (FOSA), 8:2 Fluorotelomer phosphate diester (8:2 diPAP), 8:2 fluorotelomer alcohol (8:2 FTOH), 10:2 fluorotelomer alcohol (10:2 FTOH), and 6:2 fluorotelomer sulfonamide alkylbetaine (6:2 FTAB), were compared across multiple species and systems. The approach demonstrates how comparisons of metabolic maps across species are aided by considering the sample matrix in which metabolites were quantified for each species, differences in analytical methods used to identify metabolites in each study, and the relative amounts of metabolites quantified. Overall, the pathways appear to be well conserved across species and systems. For PFAS lacking a fish metabolism study, a composite map consisting of all available maps would serve as the best basis for metabolite prediction. This emphasizes the importance and utility of collating metabolism into a searchable database such as that created in this effort.

## 1. Introduction

Perfluoroalkyl and polyfluoroalkyl substances (PFAS) have become a major focus of worldwide concern by the international regulatory community, as reflected in an explosion of recent scientific literature. PFAS are a class of very persistent structures also known as “forever chemicals” based upon the strength of the carbon–fluorine interaction, the strongest bond known to chemistry [1]. These chemicals possess unique structural attributes and function as surfactants that resist heat, oil, stains, grease, and water [2,3]. They are therefore used as fabric protectants, heat resistant non-stick cookware, adhesives, food packaging, electroplating, and aqueous fire-fighting foams [3,4,5,6,7,8,9]. As a result, PFAS are found all over the planet, do not break down in the environment, can move through soils and contaminate drinking water sources, and build up (bioaccumulate) in humans and wildlife. Toxicological concerns include various cancers; impacts on growth and development, reproduction, and thyroid functions; and impairment of both the immune system and liver function [10,11,12].

There are many challenges involved in predicting the exposure pathways of chemicals from the environment into the organism, including accounting for environmental degradation and biotransformation and understanding their effects. For methodologically challenging chemicals (MCCs), such as PFAS, there is considerable uncertainty regarding their fate and transport, biotransformation and bioaccumulation that can hamper efforts to integrate exposure and effects models.

While the perfluoroalkylcarboxylic acids and sulfonic acids are persistent chemicals presumed not capable of further biotransformation, there is much to be learned about the PFAS precursors that may eventually break down to the perfluoroalkyl acids. Transitional metabolites and their rate of formation potentially play a large role in risk assessment evaluation, for example, if the intermediates happen to exhibit greater toxicological effects. Progress in this area relies first on developing an understanding of metabolism across species for PFAS where the data exists, allowing for eventual extrapolation of concepts and lessons learned regarding PFAS and species for which little data exists. Therefore, a curation of PFAS biotransformation data from the literature is a first step. Pathways from all taxa and degradation studies were collected and coded into a MetaPath database for comparison across species/systems.

The described research has the fundamental objective of expanding the understanding of biotransformation of PFAS by fish and potentially other non-mammalian taxa, with relevance to ecological risk assessment. Understanding xenobiotic metabolism, both in terms of detoxification and bioactivation influencing exposure and effect characterizations, is a critical aspect of species extrapolation for assessing ecological risk. The role of metabolism in chemical detoxification and elimination is important as it influences chemical bioaccumulation. This research seeks to characterize the metabolites formed and compare metabolism pathways across species to better understand similarities and differences in biotransformation reactions that may lead to enhanced toxicity. Results from these efforts can then serve as a basis for predicting metabolism for untested species. Where rules can be formulated for cross-species metabolism based on transformation types leading to enhanced toxicity or detoxification, predictions of altered susceptibility to toxic events can be used to better evaluate risk where empirical data is lacking.

Limited availability of fish metabolic pathways for PFAS can result in risk assessments with inherent uncertainties based upon the assumption of the presence of only the parent chemical or the assumption that the environmental biodegradation or mammalian metabolism map data will serve as an adequate surrogate for metabolism products in fish. A database of environmental degradates and mammalian metabolic maps was systematically compared to the available fish metabolism data found in the open literature for PFAS classes. Assessment of similarities and differences in biotransformation products determined in fish can then be compared to documented pathways in mammalian and biodegradation studies for the purpose of advancing species extrapolation in efforts to reduce ecological risk assessment uncertainties.

## 2. Methods

### 2.1. Metabolic Map Collection

Initial efforts focused on open literature searches for references that include biotransformation maps inclusive of PFAS metabolism and degradation in various species and environmental systems. PubMed (https://pubmed.ncbi.nlm.nih.gov/, accessed on 6 January 2023) searches were performed using the keywords PFAS, perfluoro, polyfluoro AND metabolism, biodegradation, biotransformation, metabolite, degradate. In addition, a systematic search of the ECOTOX Knowledgebase [13] was conducted to explore specifically for PFAS biotransformation information.

An existing knowledgebase platform, introduced as MetaPath [14], for building metabolism databases with the addition of efficient and targeted tools for analyzing metabolism data, was used in this project. MetaPath was used for the curation of not only the metabolic pathways but also the supporting metadata describing study details found in the publications. A systematic process for collecting and compiling metabolism map information was developed for entry of data into MetaPath. Briefly, the process started with data entry into a standardized template called the Data Evaluation Record (DER) Composer. The standalone software template was originally designed for entry of data from registrant submitted rat and livestock studies that follow internationally coordinated pesticide metabolism data guidelines. In the work reported here, the template was used for the entry of general biotransformation studies as found in the open literature. MetaPath and DER Composer were developed by the United States Environmental Protection Agency (USEPA), Office of Research and Development (ORD), Great Lakes Toxicology and Ecology Division (GLTED) and the Laboratory of Mathematical Chemistry (LMC), Bourges, Bulgaria in partnership with USEPA, Office of Pesticide Programs (OPP). The DER Composer generates an xml file of the data that allows for the capture of the metabolism map as structures with assigned connectivity, resulting in depiction as a map or pathway in MetaPath. Additionally, the critical meta-data associated with each map to understand the origin of the data, how it was collected, including analytical chemistry techniques used to identify metabolites, the experimental design information, references, etc., is also captured. MetaPath and Composer software are open to the public and available free of charge from the developer (LMC) through download (https://oasis-lmc.org/products/software/metapath.aspx, accessed on 6 January 2023).

### 2.2. Data Quality Assurance (QA)

Quality Assurance aspects of data entry, review and tracking are addressed using a systematic process that was developed [14] in conjunction with OPP and updated in MetaPath data entry pilots with Canada’s Pesticide Management Regulatory Authority (PMRA) and France’s pesticide authority Agence nationale de sécurité sanitaire de l’alimentation, de l’environnement et du travail (ANSES). Briefly, the process goes from initial data source identification through data entry (curation), creation of xmls, and uploading of reviewed and accepted xmls into MetaPath. The final step is verification of metabolite structures in the map display and the metadata entered in MetaPath. This process results in quality assured xmls. This process includes aspects such as:

- Tracking of the data source in files and folders with systematic file naming;
- Checklists/How To guidance for initial coders;
- QA accuracy checklists for reviewers of the initial coded xml and the final MetaPath pathway.

### 2.3. Analysis of Metabolic Maps

The chemical structure search feature of MetaPath was used to determine the most common metabolites arising from maps of different parent chemicals. Exact structure and product parameter search queries for PFOA and PFOS, the most common of PFAS structures, were created and applied to the entire database of metabolic maps.

Comparisons of metabolic maps generated from different references, different parent compounds, different species, etc., can easily be made using MetaPath software. Two types of comparisons are possible in MetaPath: Direct Map Comparison and Map Similarity. The Direct Map Comparison feature involves user selection of two identified maps within the database to compare. The Map Similarity function requires the user to select and upload a map into the query. That map is then compared to all other maps within the database for metabolite commonality.

The Highlight Treatment Group is an on-screen function to provide immediate results, mapping metabolites to specific treatment groups. Treatment groups are defined as compartments isolated for analysis in a typical study. These include the matrix (i.e., blood, liver, muscle, etc.), gender (male or female), dose route, dose amount, temperature, etc., which are broken down into unique study parameters.

### 2.4. Automated PFAS Pipeline Profiler

An automated pipeline profiler was built for the classification of PFAS based upon chemical structure. The automated profiler was built upon the Pipeline Profiler ver. 1.0.52.19 software as developed by LMC (Bourgas, Bulgaria). The profiler is a dendroid logic scheme based upon a series of nodes. Each node proposes a yes/no query regarding a structural attribute. If the query is satisfied, the structure could adequately be classified or at least moved on in the direction of the affirmative path. If the query is negative, then the system moves the target structure on to the negative path. The system design is dynamic in that as new inventories of PFAS are encountered, new structural classifications may arise, resulting in an expansion of the system. A potential release of the Automated PFAS Pipeline Profiler is under discussion with the developers of the QSAR Toolbox application.

## 3. Results

### 3.1. PFAS Biotransformation Database

The compiled database specific to PFAS compounds includes over 300 maps originating from the peer reviewed literature. The species or test systems proved to be very diverse. Fish species covered in the database include rainbow trout, perch, carp, medaka, zebrafish, loach, gudgeon, barbel, turbot, and gilthead bream. Mammalian species include human, monkey, dog, sheep, pig, rat, mouse, beluga whale, ringed seal, and polar bear. Metabolic maps for the plant species carrot, wheat, pumpkin, alfalfa, soybean, lettuce, radish, mung bean, corn, and ryegrass were found. Maps for other species, such as mussels, earthworms, and chicken, are also provided. Biodegradation was reported in aerobic and anaerobic soil, sediment, and wastewater treatment plant sludge as well as bacterial and fungal microbes.

**Figure 1:**
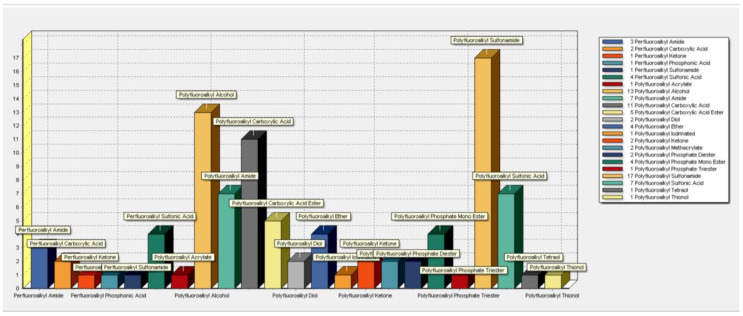
Classification of 97 PFAS found in the open literature biotransformation database, utilizing the Automated PFAS Pipeline Profiler.

The database contains 97 different PFAS structures of various per- and poly-fluoroalkyl chain lengths with diverse associated functional groups (Figure 1). The classification showed that polyfluoro- structures predominated with representation by 81 chemicals and only 16 perfluoro-structures. The major classes represented in the database were polyfluoroalkyl sulfonamides (17 structures), polyfluoroalkyl alcohols (13), polyfluoroalkyl carboxylic acids (11), polyfluoroalkyl amides (7), and polyfluoroalkyl sulfonic acids (7).

The PFAS MetaPath database is available for download as a Supplementary Material.

### 3.2. Terminal Product(s) Observed in PFAS Pathways

**Figure 2:**
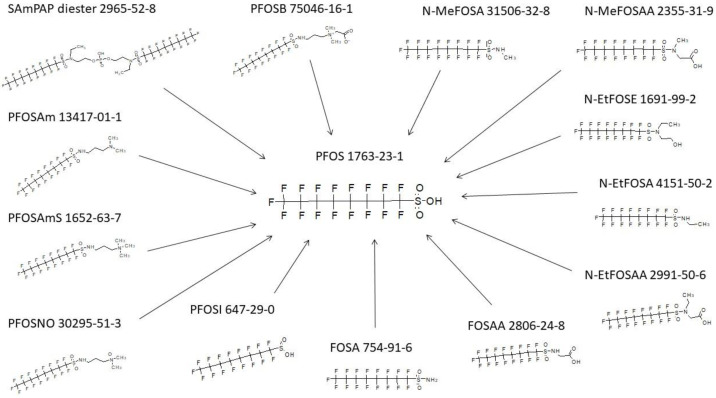
PFOS originating from a very diverse set of parent structures as found in the database.

**Figure 3:**
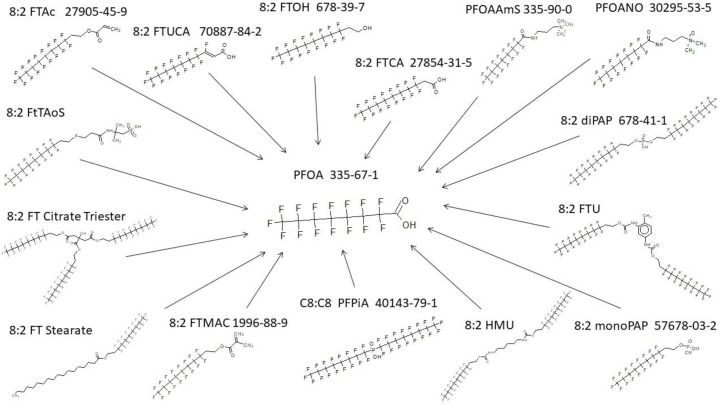
PFOA originating from a very diverse set of parent structures as found in the database.

It is thought that the biotransformation of most PFAS ultimately result in terminal perfluoroalkyl-sulfonic acid or carboxylic acid products [15]. An exact structure search for PFOS, the most prevalent of the perfluoroalkyl sulfonic acids, was performed across all the metabolic products in the complete database of 302 maps. A rather diverse set of parent structures give rise to PFOS as the end product (Figure 2). There are often multiple steps in the biotransformation, and the intermediate metabolites between parent and PFOS are not shown in the figure. PFOS has not been shown to undergo further metabolism as evidenced by the search conducted here on the literature and resultant maps. In addition to PFOS, a search for PFOA was run across the entire inventory of maps contained within the database. The results are shown in Figure 3 and depict a diverse group of parent structures. Intermediate structures found in multi-step pathways are not shown. As with the perfluoroalkyl sulfonic acids, the perfluoroalkyl carboxylic acids do not undergo any further biotransformation.

### 3.3. Evaluation of Metabolic Maps

**Figure 4:**
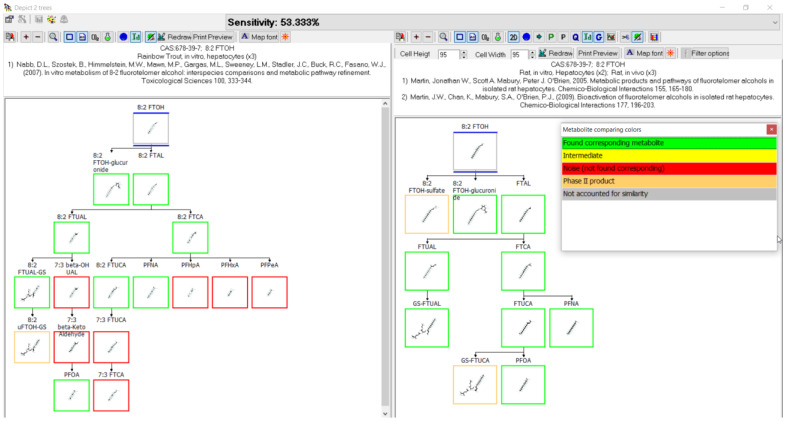
Metabolic map comparison of 8:2 FTOH in (**left**) rainbow trout hepatocytes and (**right**) rat hepatocytes. Metabolites common to both species are indicated by green boxes. Differences in metabolites are indicated by red boxes. Tan boxes indicate phase II conjugates. Sensitivity, in this case 53.333%, is an assessment of the map comparison whereby the number of common metabolites is divided by the total number of metabolites.

The following demonstrates outputs from MetaPath for displaying and comparing metabolic maps. An example of the comparison between rainbow trout and rat 8:2 FTOH metabolic maps is shown in Figure 4. Both studies were conducted using isolated hepatocytes, rainbow trout on the left and rat on the right. Green boxes represent those metabolites common to both species, red boxes indicate differences, and the tan depict phase II conjugates. The map comparison tools were used to build the composite maps portrayed in the subsequent individual chemical comparison assessment sections.

**Figure 5:**
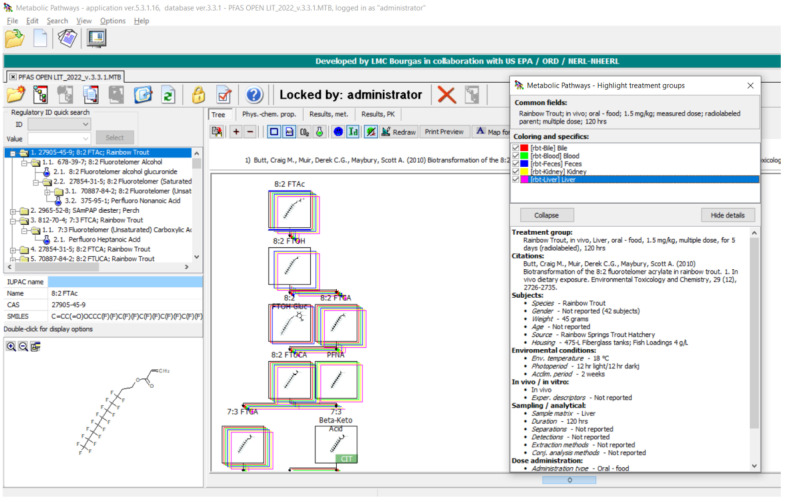
Highlight Treatment Group function for the in vivo study of 8:2 Fluorotelomer Acrylate (8:2 FTAc) in rainbow trout. Colored boxes around metabolites in the metabolic map are correlated to the respective treatment groups, which are bile, blood, feces, kidney, and liver, in this case.

An example of the Highlight Treatment Group is shown in Figure 5. This functionality enabled an immediate and a more detailed assessment of metabolite distribution in distinct sample matrices as defined by treatment groups.

The PFAS metabolic maps assembled and constructed from the open literature indicated multiple biotransformation pathways shared across species. The following section provides demonstration, comparison, and comment on biotransformation of 10 PFAS structures.

### 3.4. C6/C8 PFPiA Biotransformation

**Figure 6:**
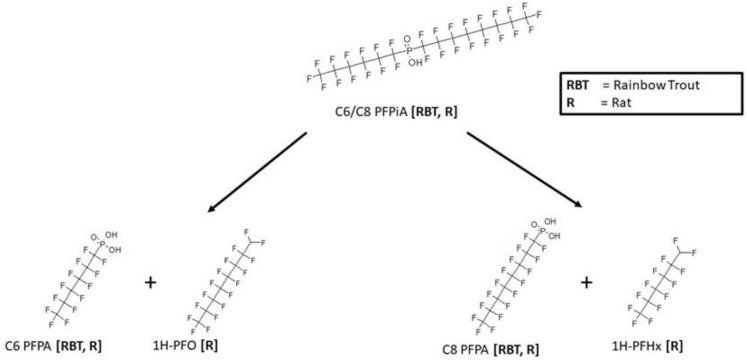
Metabolic map for heptadecafluorooctyl(tridecafluorohexyl)phosphinic acid (C6/C8 PFPiA) as found in rainbow trout [16] and rat [17].

Metabolic maps for the metabolism of perfluoro-hexyl-octyl-phosphinic acid were found in the rainbow trout [16] and rat [17]. Both were in vivo studies that resulted in a cleavage of parent to yield either the perfluorohexyl- or perfluorooctyl-phosphonic acid (Figure 6). Rainbow trout samples were the whole animal homogenates, while the blood and liver were analyzed in the rat. In addition, the rat study also showed metabolites 1H-perfluorooctane or 1H-perfluorohexane that were complimentary to the respective phosphonic acid products.

### 3.5. C6/C6 PFPiA and C8/C8 PFPiA Biotransformation

**Figure 7:**
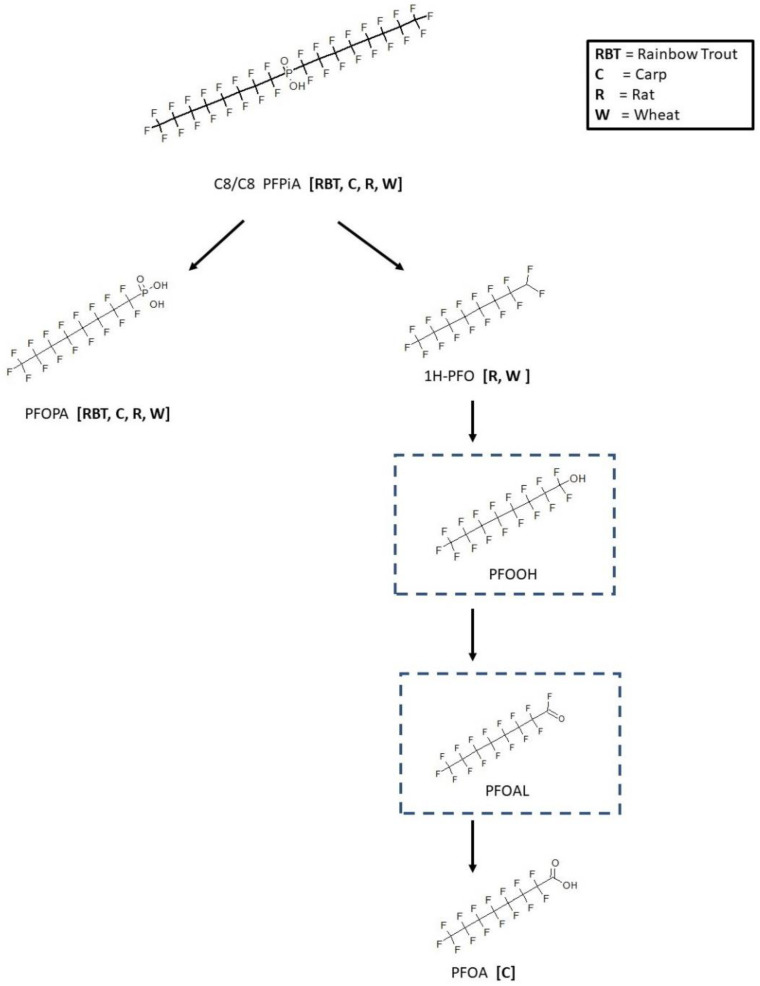
Metabolic map for bis(perfluorooctyl)phosphinic acid (C8/C8 PFPiA) as found in rainbow trout [16], rat [17], carp [18], and wheat [19]. Structures as shown within the dotted box were assumed by the authors but were not actually observed metabolites in the studies.

Metabolic maps for the symmetrical analogues bis(perfluorohexyl)phosphinic acid (C6/C6 PFPiA) and bis(perfluorooctyl)phosphinic acid (C8/C8 PFPiA) were also available. The maps for C6/C6 and C8/C8 PFPiA resulted in similar metabolic patterns. The pathway for rainbow trout [16], rat [17], carp [18], and wheat [19] is shown in Figure 7. The rainbow trout samples were whole animal homogenates, while blood and liver were analyzed in the rat from in vivo chemical exposures. The carp study was an in vivo exposure with analysis of blood, brain, bile, intestine, kidney, liver, gill, and muscle tissues. All species demonstrated an enzymatic cleavage reaction of parent chemical resulting in the identification of perfluorooctylphosphonic acid (PFOPA). PFOPA was found in the bile, intestine, kidney, and liver of carp. The other product, 1H-perfluorooctane, resulting from the cleavage reaction was observed in only the rat and wheat maps. 1H-perfluorooctane and PFOPA were found in both the roots and shoots of wheat. Perfluorooctanoic acid (PFOA) was only found in the carp liver and is presumed to have come from the sequential oxidation of 1H-perfluorooctane to the perfluorooctyl alcohol, then to the perfluorooctyl aldehyde, and finally to PFOA.

### 3.6. 6:2 Cl-PFESA and 8:2 Cl-PFESA Biotransformation

**Figure 8:**
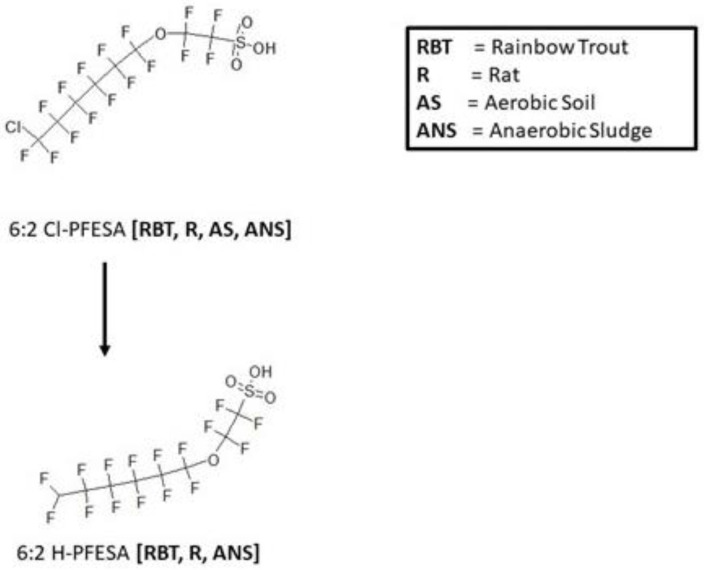
Metabolic map for 2-[(6-chloro-1,1,2,2,3,3,4,4,5,5,6,6-dodecafluorohexyl)oxy]-1,1,2,2-tetrafluoroethanesulfonic acid (6:2 Cl-PFESA) as found in rainbow trout [20], rat [21], aerobic soil [22], and anaerobic sludge [23].

A metabolic map for 2-[(6-chloro-1,1,2,2,3,3,4,4,5,5,6,6-dodecafluorohexyl)oxy]-1,1,2,2-tetrafluoroethanesulfonic acid (6:2 Cl-PFESA) in rainbow trout [20], rat [21], aerobic soil [22], and anaerobic sludge [23] is shown in Figure 8. The rainbow trout (blood, liver, kidney) and rat (urine, blood, fat, heart, kidney, liver, muscle) were in vivo exposure studies. The parent chemical, 6:2 Cl-PFESA, undergoes a reductive dechlorination reaction to form 6:2 H-PFESA microbially in anaerobic sludge. The metabolite 6:2 H-PFESA was also found in all matrices of the rainbow trout and rat. In contrast, however no microbial metabolism of 6:2 Cl-PFESA was observed in aerobic soil samples.

### 3.7. N-EtFOSA Biotransformation

**Figure 9:**
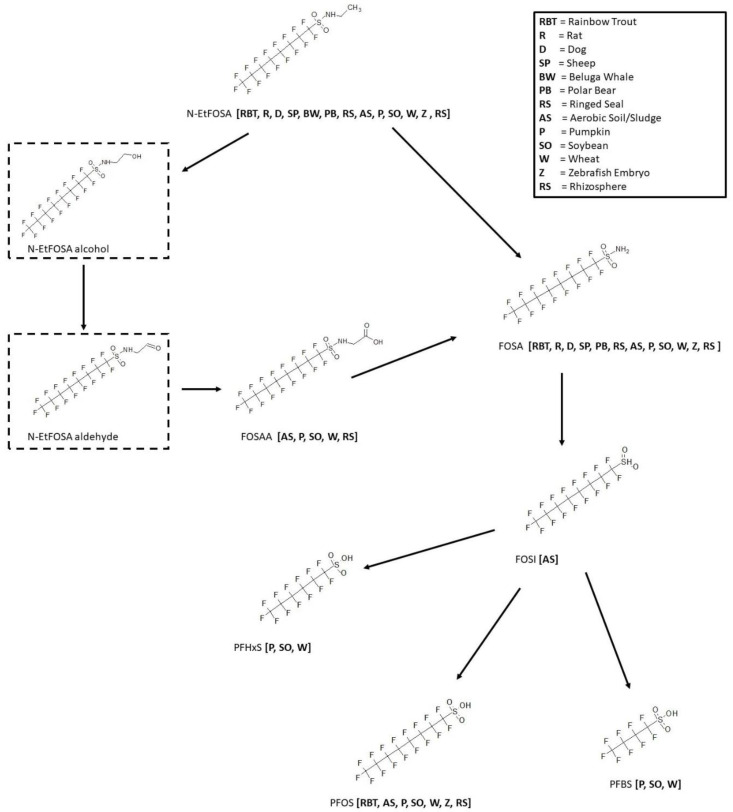
Metabolic map for *N*-Ethylperfluorooctane-1-sulfonamide (Sulfuramid; N-EtFOSA) as found in rainbow trout [24], rat [25,26,27,28], dog [27], sheep [29], beluga whale [26], polar bear [26], ringed seal [26], aerobic soil/sludge [31,32], pumpkin [34], soybean [34], wheat [34], zebrafish embryo [30], and rhizospheres [33]. Structures as shown within the dotted box were assumed by the authors but were not actually observed metabolites in the studies.

The biotransformation of N-ethylperfluorooctane-1-sulfonamide (N-EtFOSA), also known as the insecticide sulfuramid, has been extensively studied among a wide variety of species (Figure 9). In vitro microsomal studies were conducted in the rainbow trout [24], rat [25,26], beluga whale [26], polar bear [26], and ringed seal [26]. In vivo exposure studies were performed in the rat [27,28], dog [27], sheep [29], and zebrafish embryo [30]. N-EtFOSA was shown to undergo N-dealkylation to form FOSA in all species studied except for the beluga whale. It is assumed the FOSA then goes through an intermediate sulfinic acid, only seen in aerobic soil samples, and onto PFOS as a final product. PFOS was observed in the rainbow trout, aerobic soil [31,32], pumpkin, soybean, wheat, zebrafish embryo, and rhizospheres [33]. Additional sulfonic acids, perfluorohexyl (PFHxS) and perfluorobutyl (PFBS) were observed in the pumpkin [34], soybean [34], and wheat [34] plant experiments. As part of the metabolic pathway, N-EtFOSA alcohol and N-EtFOSA aldehyde are proposed intermediates to eventually form FOSAA in the aerobic soil, pumpkin, soybean, wheat and rhizospheres. These intermediates were not actually found in the studies but were proposed by the authors and are indicated in the figure by dashed-line boxes.

### 3.8. SAmPAP Diester Biotransformation

**Figure 10:**
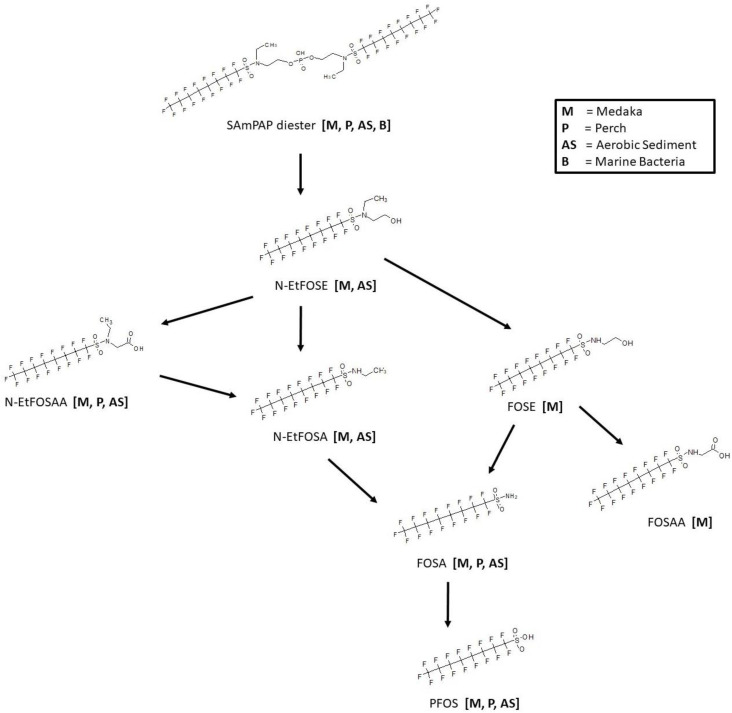
Metabolic map for SAmPAP diester as found in medaka [35], perch [36], aerobic sediment [37], and marine bacteria [38].

Studies are available for the biotransformation of SAmPAP diester in medaka [35], perch [36], aerobic sediment [37], and marine bacteria [38] (Figure 10). A complete metabolic pathway has been shown for a medaka in vivo study of whole fish homogenate whereby the SAmPAP diester is cleaved to form N-EtFOSE. The N-EtFOSE is then subjected to either oxidation of the alcohol moiety to form N-EtFOSAA or an N-dealkylation to form N-EtFOSA and FOSE. Further N-dealkylation of both N-EtFOSA and FOSE produces the sulfonamide FOSA. The final step in the map is conversion of FOSA to PFOS. In addition, FOSAA was produced by oxidation of the alcohol FOSE. The pathway from SAmPAP diester to PFOS was conserved for in vivo perch liver, serum, and muscle samples. The degradation of SAmPAP to PFOS was found in aerobic soil and presumed to transform through the observed intermediates N-EtFOSE, N-EtFOSAA, N-EtFOSA, and FOSA. In contrast, there was no observed biotransformation of SAmPAP diester in the presence of marine bacteria.

### 3.9. FOSA Biotransformation

**Figure 11:**
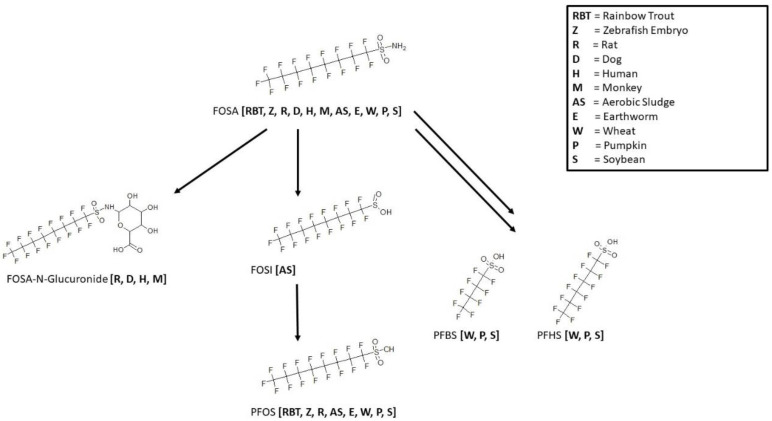
Metabolic map for Perfluorooctanesulfonamide (FOSA) as found in rainbow trout [39], rat [25,40], dog [40], human [40], monkey [40], aerobic sludge [32], earthworm [41,42], pumpkin [43], soybean [43], wheat [41], and zebrafish embryo [30].

Consistent with the observed process in the overall map for biotransformation of SAmPAP diester (Figure 10), the conversion of FOSA to PFOS was observed in rainbow trout [39], zebrafish embryo [30], rat [25,40], earthworm [41,42], aerobic soil [32], wheat [41], pumpkin [43], and soybean [43] (Figure 11). The muscle of rainbow trout was analyzed following in vivo exposure. Zebrafish embryo and earthworm were exposed in vivo and sampled as whole animal extractions. The first rat study was conducted in vitro with liver microsomes, cytosol, and tissue slices. The sulfinic acid FOSI is a proposed intermediate but was only observed in aerobic soil samples. The parent chemical, FOSA, was shown to form a glucuronide conjugate in the microsomes of dog [40], rat [40], human [40], and monkey [40] in the presence of cofactors for UDP-glucuronosyltransferase activity. In addition to PFOS, the shorter chain perfluoroalkyl sulfonic acids, PFHxS and PFBS, were exclusively found in the plant species maps for wheat, pumpkin, and soybean.

### 3.10. 8:2 diPAP Biotransformation

**Figure 12:**
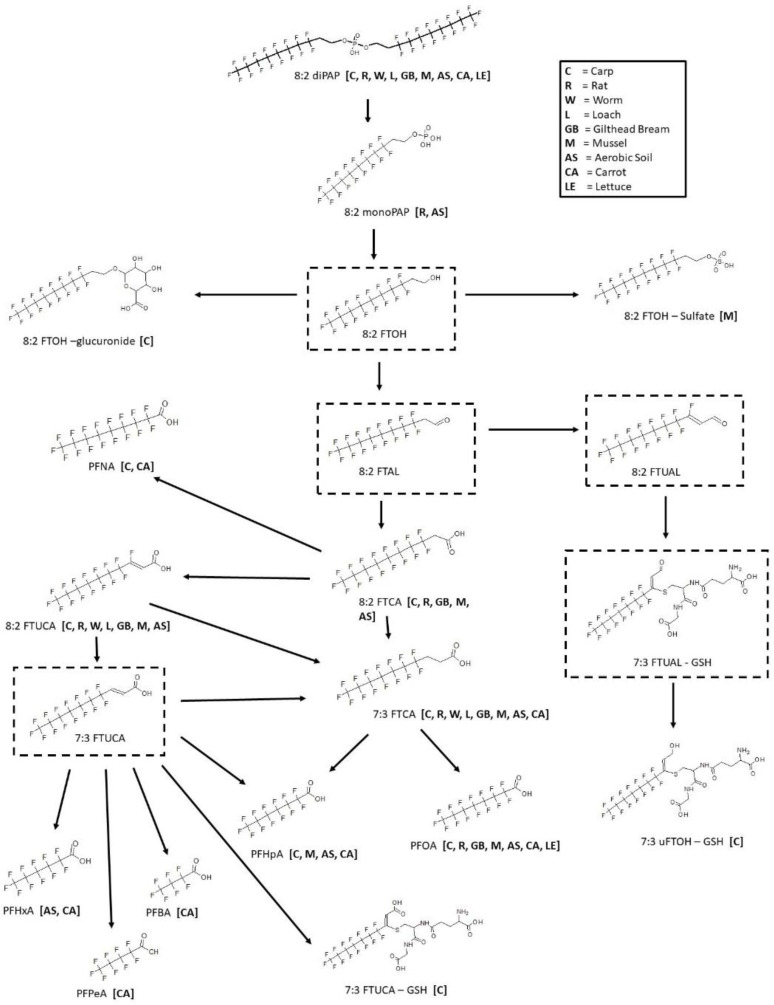
Metabolic map for 8:2 Fluorotelomer phosphate diester (8:2 diPAP) as found in carp [44,45], rat [46], worm [44], loach [44], gilthead bream [47], mussel [48], aerobic soil [49,50], carrot [50], and lettuce [50]. Structures as shown within the dotted box were assumed by the authors but were not actually observed metabolites in the studies.

Metabolism of the 8:2 fluorotelomer phosphate diester was studied in carp [44,45], rat [46], worm [44], loach [44], gilthead bream [47], mussel [48], aerobic soil [49,50], carrot [50], and lettuce [50] (Figure 12). The carp and loach studies were both in vivo with whole fish extracts. Gilthead bream, also in vivo, examined bile, brain, gills, liver, muscle and plasma for metabolites. The rat was also an in vivo study with the analysis of blood, brain, fat, kidney, liver, muscle, spleen, and testes. Whole worm extracts and the digestive gland, gills, gonad, mantle, muscle, feces, and elimination water of mussel in vivo studies were analyzed for metabolites. The pathway assumes a hydrolysis of the diester phosphate to the monophosphate ester as found in the rat and aerobic soil samples. The monophosphate ester is then presumed to proceed through the alcohol, 8:2 FTOH. Although not actually observed in any of the sample matrices, the presence of an 8:2 FTOH intermediate is supported by the presence of conjugates 8:2 FTOH-glucuronide in carp and 8:2 FTOH-sulfate in mussel. The 8:2 FTOH then undergoes oxidation to another presumed intermediate, 8:2 aldehyde, and then the carboxylic acid 8:2 FTCA as found in carp, rat, gilthead bream, mussel, and aerobic soil. Other fluorotelomer carboxylic acids found in the pathway include 8:2 FTUCA as found in carp, rat, worm, loach, gilthead bream, mussel, aerobic soil and 7:3 FTCA as found in carp, rat, worm, loach, gilthead bream, mussel, aerobic soil, and carrot. In addition, although not found, 7:3 FTUCA is a presumed intermediate. Several perfluorocarboxylic acids were found among and across various studies. Perfluorononanoic acid (PFNA) was found in carp and carrot while perfluorooctanoic acid (PFOA) was found in carp, rat, gilthead bream, mussel, aerobic soil, lettuce, and carrot. Perfluoroheptanoic acid (PFHpA) was found in carp, mussel, aerobic soil, and carrot, while shorter chain perfluorohexanoic acid (PFHxA) was found in aerobic soil and carrot and both perfluoropentanoic acid (PFPeA) and Perfluorobutanoic acid (PFBA) were found in carrot. Glutathione conjugates of the 7:3 fluorotelomer unsaturated carboxylic acid (7:3 FTUCA-GSH) and 7:3 fluorotelomer unsaturated alcohol (7:3 uFTOH-GSH) were both found in the carp samples.

### 3.11. 8:2 FTOH Biotransformation

**Figure 13:**
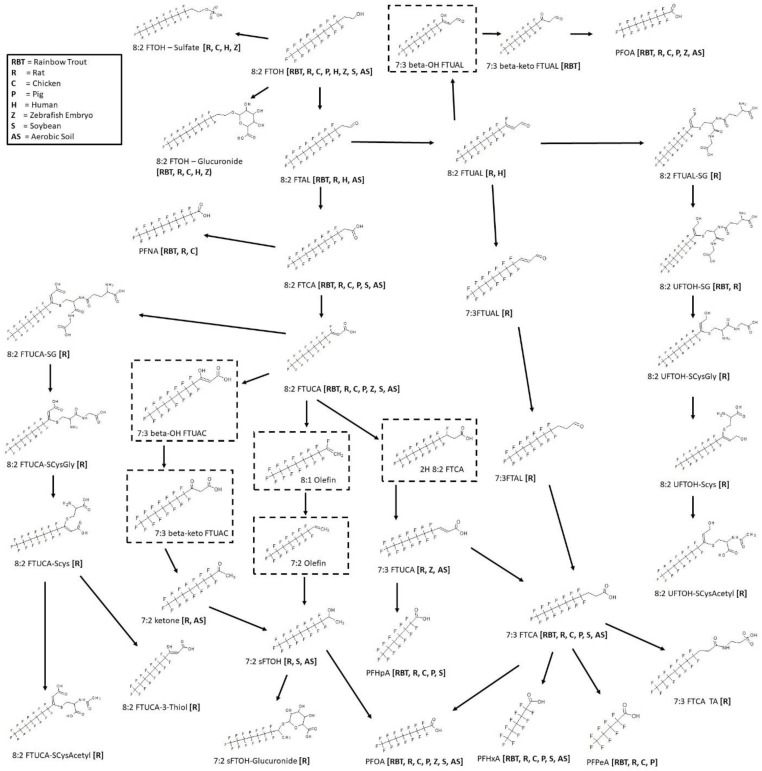
Metabolic map for 8:2 fluorotelomer alcohol (8:2 FTOH) as found in rainbow trout [39,51], rat [52,53,54,55,56], chicken [57], pig [58], human [59], zebrafish embryo [30], soybean [60], and aerobic soil [61,62]. Structures as shown within the dotted box were assumed by the authors but were not actually observed metabolites in the studies.

Biotransformation was reported for the 8:2 fluorotelomer alcohol (8:2 FTOH) in rainbow trout [39,51], rat [52,53,54,55,56], chicken [57], pig [58], human [59], zebrafish embryo [30], soybean [60], and aerobic soil [61,62] (Figure 13). There were two studies contributing to the rainbow trout map, in vivo where muscle was analyzed for metabolites and in vitro with isolated hepatocytes. Five different in vivo rat studies were conducted with the analysis of urine, feces, bile, plasma, blood, kidney, liver, skin, bone marrow, fat, and thyroid. An additional in vitro rat study with isolated hepatocytes was conducted. Chicken and pig studies were done in vivo and the fat, heart, kidney, liver, lung, and muscle were analyzed for metabolite formation. Whole embryo extracts were studied in zebrafish in vivo experiments. Human studies were in vitro with cytosol, microsomes, and individual CYP’s. As in the 8:2 diPAP map (Figure 12), conjugates for 8:2 FTOH were reported as 8:2 FTOH-sulfate in rat, chicken, human, and zebrafish and 8:2 FTOH-glucuronide in rainbow trout, rat, chicken, human, zebrafish. The rainbow trout pathway goes through oxidation of 8:2 FTOH to the identified 8:2 fluorotelomer aldehyde (8:2 FTAL), the carboxylic acid (8:2 FTCA), and unsaturated acid (8:2 FTUCA). The 7:3 fluorotelomer carboxylic acid (7:3 FTCA) was also identified. The series of perfluoroalkyl carboxylic acids were found in rainbow trout: PFNA, PFOA, PFHpA, PFHxA, and PFPeA. Most of these terminal products were also found in rat, chicken, pig, zebrafish, and aerobic soil. The rat map contributed an extensive list of intermediates and various conjugates with glutathione, cysteinylglycine, cysteine, cysteineacetyl, thiol, and taurine.

### 3.12. 10:2 FTOH Biotransformation

**Figure 14:**
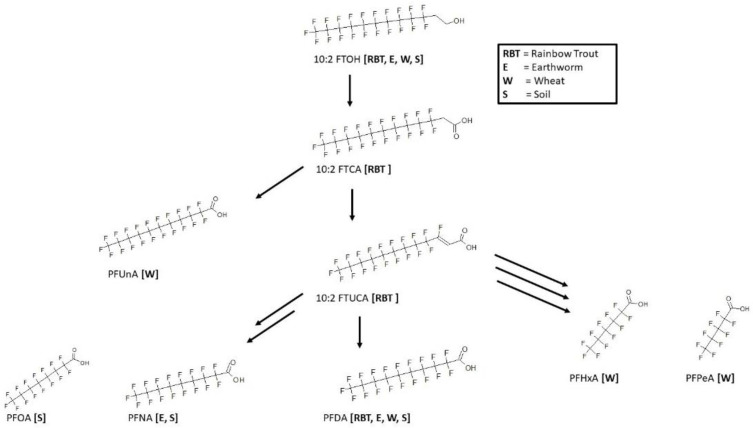
Metabolic map for 10:2 fluorotelomer alcohol (10:2 FTOH) as found in rainbow trout [39], earthworm [63], wheat [63], and soil [63].

Metabolic maps for 10:2 fluorotelomer alcohol (10:2 FTOH) were determined for rainbow trout [39], earthworm [63], wheat [63], and soil [63] (Figure 14). The rainbow trout and earthworm were both in vivo studies whereby the trout muscle and whole worm homogenate were analyzed for metabolites. In rainbow trout, the 10:2 FTOH has been shown to metabolize through the fluorotelomer carboxylic acid, 10:2 FTCA and fluorotelomer unsaturated carboxylic acid, 10:2 FTUCA on route to the final product perfluorodecanoic acid (PFDA). In the other species, only the final perfluoroalkyl acids were found. In earthworm, PFNA was found in addition to PFDA. Soil samples contained PFOA, PFNA, and PFDA. Wheat samples of the root and shoots contained the most extensive group of perfluoroalkyl acids: perfluoroundecanoic acid (PFUnA), PFDA, PFHxA, and PFPeA.

### 3.13. 6:2 FTAB Biotransformation

**Figure 15:**
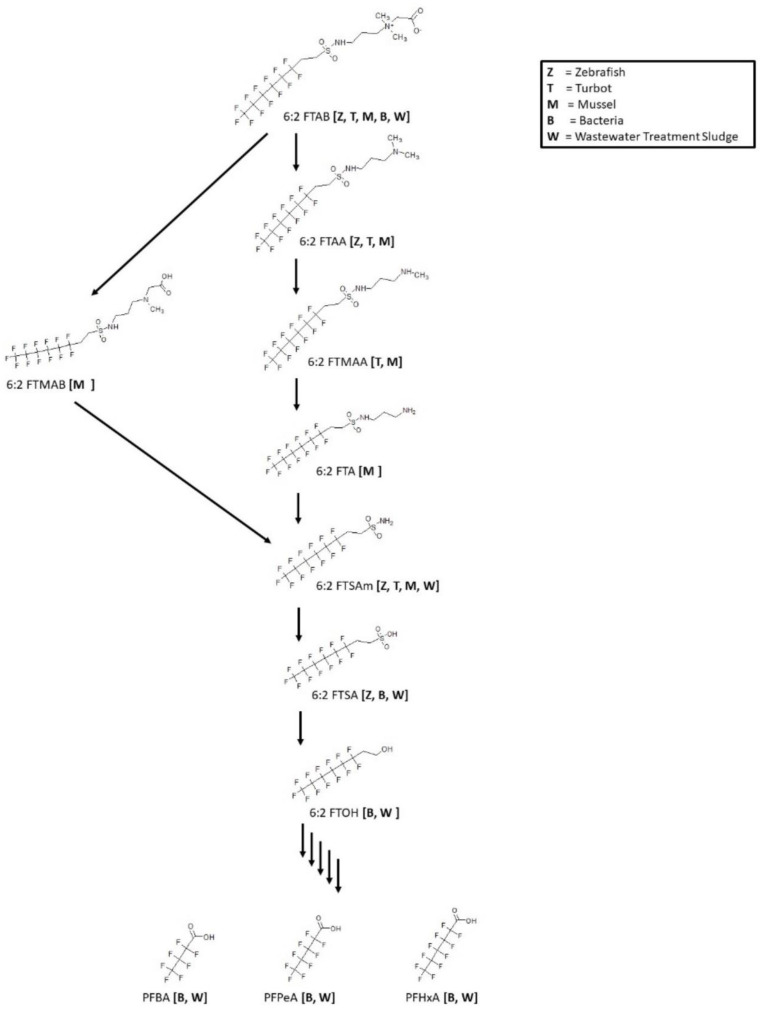
Metabolic map for 6:2 fluorotelomer sulfonamide alkylbetaine (6:2 FTAB) as found in zebrafish [64], turbot [65], mussel [65], bacteria [66] and wastewater treatment sludge [67].

The surfactant, 6:2 fluorotelomer sulfonamide alkylbetaine (6:2 FTAB), a novel PFOS alternative is used globally in aqueous film forming foams (AFFFs). Biotransformation studies were reported for zebrafish [64], turbot [65], mussel [65], bacteria [66] and wastewater treatment sludge [67] (Figure 15). The zebrafish, turbot, and mussel studies were all in vivo experiments. Whole mussel homogenates were extracted; turbot liver and zebrafish liver, testes, ovaries, and embryos were analyzed. The consensus metabolic pathway for 6:2 FTAB goes through multiple N-dealkylation steps down to the sulfonamide 6:2 FTSAm. The primary sulfonamide is then subjected to a deamination step to produce the sulfonic acid 6:2 FTSA. Reactions of bacteria and wastewater treatment sludge were shown to further degrade the 6:2 FTSA to 6:2 FTOH. The 6:2 FTOH further broke down to perfluoro-carboxylic acids as indicated by the presence of PFHxA, PFPeA, and PFBA in bacteria and wastewater treatment sludge experiments.

## 4. Discussion

The process presented herein represents a rapid and transparent approach to comparing metabolisms across species through the compilation of a PFAS biotransformation database and then using MetaPath and associated evaluation tools to facilitate the analyses.

Unlike the guideline studies associated with registrant pesticide submissions that follow a specific protocol, providing more consistency for data comparisons [68], PFAS metabolism data examined here is more variable with respect to the biological model and experimental design. The previous work involved a species comparison of a fish study associated with bioaccumulation limited to bluegill or rainbow trout with rat metabolism and goat livestock residue studies. This recent effort resulted in the MetaPath database compilation of PFAS biotransformation data from studies published in the open literature that included over 35 different species as well as various sediments and sludges. Not only is this a more species diverse set of maps, it also includes various levels of biological organization, i.e., in vivo studies or in vitro studies using cytosol, microsomes, S9, hepatocytes, and tissue slices. The in vivo studies examined a multitude of sample matrices, including blood, plasma, liver, kidney, heart, lung, brain, thyroid, gill, intestine, spleen, muscle, fat, whole animal, urine, feces, core, leaves, peel, root, and shoots, that were appropriately applicable to the species. Add in the manner and level of PFAS dosing with the eventual analytical chemistry methods utilized for metabolite identification and comparison of biotransformation maps can be complicated. None-the-less, the purpose of this research effort was to select 10 PFAS where available fish maps could be compared with other species or systems to evaluate their potential to serve as surrogates where the fish maps are lacking.

All taxa are exposed to potentially toxic foreign chemicals and to some extent try to metabolize those structures to either render them less toxic or to facilitate elimination. While careful not to dismiss extrahepatic metabolism, in fish, mammals, poultry, and other species where a distinct organ exists, it is the liver that functions as the primary site for that biotransformation [69,70]. Metabolic pathways generally refer to a Phase I set of reactions through cytochrome P450 transformation whereby a parent chemical is altered through an oxidation, dealkylation or hydrolysis mechanism to afford a precursor for subsequent conjugation known as Phase II. Phase II reactions include pathways, such as glucuronosyltransferase, sulfotransferase, and glutathione transferase which all render a more hydrophilic structure [71,72]. The next step in liver metabolism is often considered the actual elimination process or Phase III. By comparison, plant metabolism too functions through a set of transformation (Phase I) and conjugation (Phase II) reactions whereby the same enzymatic pathways are conserved [73]. The difference being that plants offer no true elimination pathway to serve as a Phase III path. Instead, plants tend to function through compartmentation and storage rather than elimination. Examples of the storage path are export into vacuoles, export into extracellular space, or deposition into lignin or cell wall components. Regardless of a Phase III through elimination in the liver or storage in the plant, the actual enzymatic processes of Phase I and II seem to be well conserved from plant to animal. These metabolic processes are expected to be largely conserved across microbial communities as well.

True species differences do exist in the metabolism of xenobiotics. For example, the metabolism of 2,4-dimethylaniline (2,4-DMA) was compared in vivo in the rat and dog [74]. The major metabolite found in the rat but not in the dog was N-acetyl-4-amino-3-methyl benzoic acid (AAMBA). The dog produced metabolites that were not found in the rat; a major product 6-hydroxy-2,4-dimethylaniline (6-HDMA) and a minor metabolite 4-amino-3-methyl benzoic acid (4-AMBA). Acetylation is a relatively common pathway; however, dogs are not able to acetylate N-arylamines. A species difference in metabolism can influence a significant difference in toxicity. The exposure of 2,4-DMA produces hepatic cholangiofibrosis, bile duct proliferation, and foci of cellular hyperplasia and degeneration in the rat, while being relatively non-toxic in the dog.

An apparent species difference was observed for the biotransformation of PFOSA to PFOS in three North Sea predators [75]. The profiles of several PFAS were measured in the white-beaked dolphin (*Lagenorhynchus albirostris*), the harbor porpoise (*Phocoena phocoena*), and the harbor seal (*Phoca vitulina*). When considering the ratios of PFOS/PFOSA of 766 in the harbor seal as compared to 20 in the harbor porpoise and 2.4 in the dolphin, this ratio would indicate that the harbor seal of the order Carnivora has a much higher capacity of transforming PFOSA to PFOS than the other two species that belong to the order Cetacea. This observation was supported by the relatively high PFOS/PFOSA ratio reported for other Carnivora, the polar bear [76] and ringed seal [77], while the Cetacea, bottle-nose dolphin [78] and beluga whale [79] showed relatively low PFOS/PFOSA ratios.

Comparisons across species were made for the PFAS biotransformation pathways as presented in this review paper. The intent was to define the extent that other species and systems could serve as suitable surrogates for fish metabolism of a variety of PFAS.

The PFPiA biotransformation study with the C6/C8 structure revealed the same transformation step, a cleavage of the phosphinic acid producing both a C6 and C8 phosphonic acid in both the rat and rainbow trout. The other product, while only observed in the rat study can be assumed to also occur in the C6/C8 PFPiA metabolism of the rainbow trout. When considering the biotransformation of the symmetrical analogue, C8/C8, all species (rainbow trout, carp, rat, wheat) produced the same initial cleavage reaction as observed for the C6/C8 analogue. In addition to the phosphonic acid, there is evidence for further transformation of the 1H-perfluoroalkane. PFOA was detected in the liver of carp exposed to C8/C8 PFPiA with the implied hydroxylation of 1H-perfluorooctane followed by sequential oxidation through an aldehyde. Even though not explicitly found in the rainbow trout this potential route of transformation found in another fish species seems reasonable to predict.

Biotransformation of 6:2 Cl-PFESA in the rat, rainbow trout, and anaerobic sludge involved a reductive dechlorination to form 6:2 H-PFESA. It was originally thought that the ether linkage inserted within the perfluoroalkyl chain on this PFOS alternative would facilitate the ability of the chemical to metabolize. In addition, it was assumed the substitution of a chlorine for fluorine on the structure would also lend itself to future metabolism. The ether linkage did not afford any metabolism. Further, the lack of reactivity observed for aerobic soil with dechlorination found in anaerobic sludge supports an anaerobic microbial pathway. This type of reaction could be predicted for other similar structures in the rainbow trout.

The biotransformation of N-EtFOSA in rainbow trout has been shown to go through an N-dealkylation of the ethyl group to form FOSA, which in turn forms PFOS. This pathway was conserved across the various species and systems presented in the overall map. The observed differences in the overall map were in the plant species, pumpkin, wheat and soybean, for which there were additional shorter chain perfluoroalkyl sulfonic acids not produced in fish or mammals. The FOSA pathway, which is actually a sub-map of the N-EtFOSA, revealed the same observed conversion of FOSA to PFOS for all species and systems. Again, there was no evidence for the shorter chain perfluoroalkyl sulfonic acids (PFBS and PFHxS) in mammals or fish that were found in plants, indicative of a species difference. Mammalian microsomal systems specifically designed with cofactors for glucuronidation were shown to produce a conjugate. Whether FOSA will form a glucuronide conjugate in fish will require further investigation, but the pathway from FOSA to PFOS is well conserved across species.

A complete map detailing initial cleavage of SAmPAP diester to N-EtFOSE, followed by a conversion to three intermediates, N-EtFOSAA, N-EtFOSA, and FOSE eventually converging to FOSA as a precursor to final product PFOS, was observed for Medaka. Most of the pathway was also observed in aerobic sediment, meaning the aerobic sediment map provides a good prediction for the pathway in fish. The perch study was informative in that the final product PFOS, as well as its precursor FOSA were detected. However, for perch, most of the early intermediates, as found in both medaka and sediment, were not observed experimentally. Overall metabolite quantities were low in perch. Perch were exposed through diet, in contrast to the medaka experiments through the water, thus indicating route of chemical exposure and availability is an important consideration when comparing metabolic maps.

Metabolism of the fire-fighting foam constituent 6:2 FTAB was reported in two species of fish. Results indicate a multi-step process for zebrafish and turbot from parent 6:2 FTAB to the fluorotelomer sulfonamide 6:2 FTSAm. The 6:2 FTSAm is further converted to the fluorotelomer sulfonic acid 6:2 FTSA in zebrafish but not the turbot. Maps were also available for mussel, bacteria, and wastewater sludge. The mussel metabolism compared well with the fish maps, but both bacteria and wastewater indicate a pathway beyond 6:2 FTSA to the fluorotelomer alcohol 6:2 FTOH and finally to the perfluoroalkyl carboxylic acids PFHxA, PFPeA and PFBA. Therefore, the documented pathway from 6:2 FTAB to 6:2 FTSA appears to be conserved across all species but further metabolism beyond the fluorotelomer sulfonic acid cannot be supported in fish.

The fluorotelomer alcohol, 8:2 FTOH pathway was the most complex map considered in this evaluation which featured 30 identified metabolites and another six presumed transitional intermediates assumed by the authors. The composite map was dominated by 29 of the 30 structures that are found in rat. Further, 13 of the metabolites were found exclusive to rat and 11 of those were conjugate structures. Overall, there were 14 conjugate structures. Glucuronide conjugation of 8:2 FTOH was observed in rainbow trout, rat, chicken, human, and zebrafish embryo while sulfate conjugation was found in rat, chicken, human, and zebrafish. Beyond the conjugation reactions, consider the five to nine carbon perfluoroalkyl carboxylic acids as terminal products in the biotransformation pathway. PFNA, PFOA, PFHpA, PFHxA, and PFPeA were all found in rainbow trout, rat, and chicken. The main pathways include multiple oxidation steps from fluorotelomer alcohol 8:2 FTOH through fluorotelomer aldehydes and fluorotelomer carboxylic acids, saturated and unsaturated. There is a reasonable expectation that the other species, such as rat, serve as reasonable predictors for phase I metabolism of fluorotelomer alcohols in fish. Although projecting some possible structures, the phase II conjugation reactions require further investigation to determine if the rat observations will transfer to fish.

The 8:2 fluorotelomer phosphate diester 8:2 diPAP in carp was proposed to hydrolyze to 8:2 monoPAP and 8:2 FTOH. There is indirect proof of the initial hydrolysis reaction based upon detection of 8:2 monoPAP, as found in both rat and aerobic soil. In addition, a glucuronide conjugate of 8:2 FTOH was shown in carp. The pathway follows the biotransformation of 8:2 FTOH as previously discussed. It is noteworthy to mention the identification of two glutathione conjugates as found in carp.

Metabolism of the fluorotelomer alcohol, 10:2 FTOH, as measured in rainbow trout was shown to undergo oxidation to the fluorotelomer carboxylic acid, through the fluorotelomer unsaturated carboxylic acid, and then to the final product PFDA. Earthworm, wheat, and soil studies also resulted in production of PFDA but did not identify the two intermediates 10:2 FTCA and 10:2 FTUCA. In addition, the other studies provided additional metabolites that were not observed in rainbow trout. The plant study reported the additional perfluoroalkyl carboxylic acids: PFUnA, PFHxA and PFPeA. PFNA and PFOA were found in soil and PFNA was found in earthworm. These latter studies provided little information with regard to modeling the biotransformation of 10:2 FTOH in fish using plant, soil and worm. It was previously shown that other species, such as rat, more closely supported biotransformation of fluorotelomer alcohols as observed for 8:2 FTOH.

**Figure 16:**
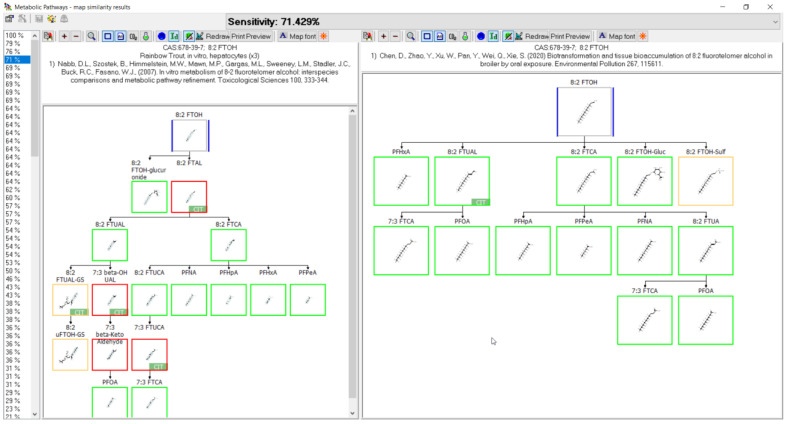
Metabolic map comparison of 8:2 FTOH in (**left**) rainbow trout hepatocytes and (**right**) in vivo chicken. Metabolites common to both species are indicated by green boxes. Differences in metabolites are indicated by red boxes. Tan boxes indicate phase II conjugates. Sensitivity, in this case 71.429%, is an assessment of the map comparison whereby the number of common metabolites is divided by the total number of metabolites in the fish map. Metabolites labeled with CIT are presumed intermediates in the path but were not identified in the study.

**Figure 17:**
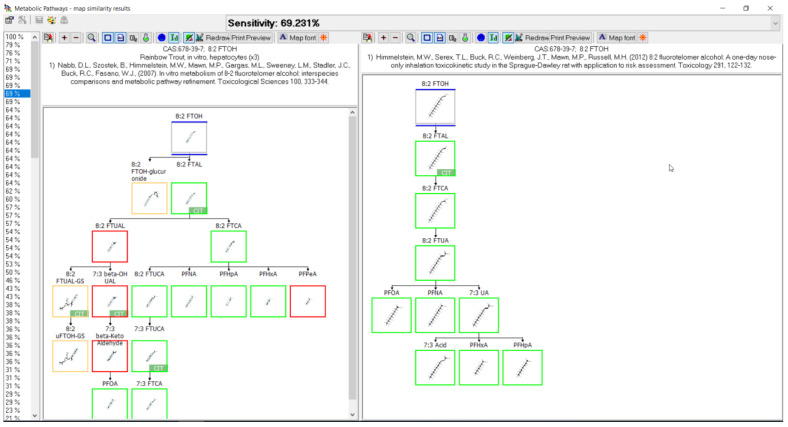
Metabolic map comparison of 8:2 FTOH in (**left**) rainbow trout hepatocytes and (**right**) in vivo rat. Metabolites common to both species are indicated by green boxes. Differences in metabolites are indicated by red boxes. Tan boxes indicate phase II conjugates. Sensitivity, in this case 69.231%, is an assessment of the map comparison whereby the number of common metabolites is divided by the total number of metabolites in the fish map. Metabolites labeled with CIT are presumed intermediates in the path but were not identified in the study.

**Figure 18:**
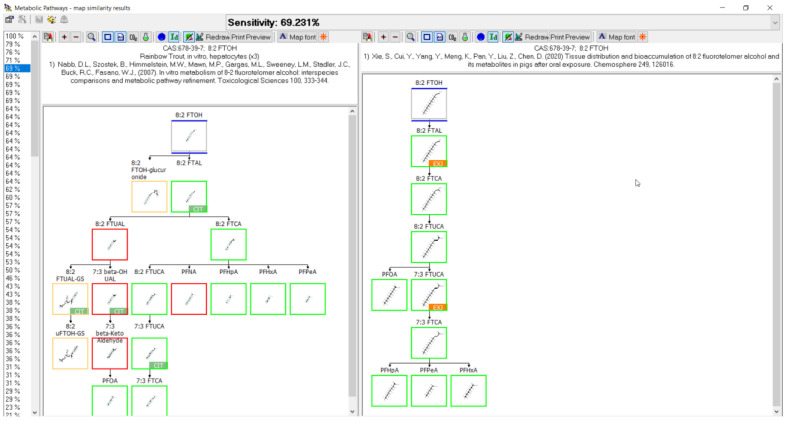
Metabolic map comparison of 8:2 FTOH in (**left**) rainbow trout hepatocytes and (**right**) in vivo pig. Metabolites common to both species are indicated by green boxes. Differences in metabolites are indicated by red boxes. Tan boxes indicate phase II conjugates. Sensitivity, in this case 69.231%, is an assessment of the map comparison whereby the number of common metabolites is divided by the total number of metabolites in the fish map. Metabolites labeled with CIT are presumed intermediates in the path but were not identified in the study.

**Figure 19:**
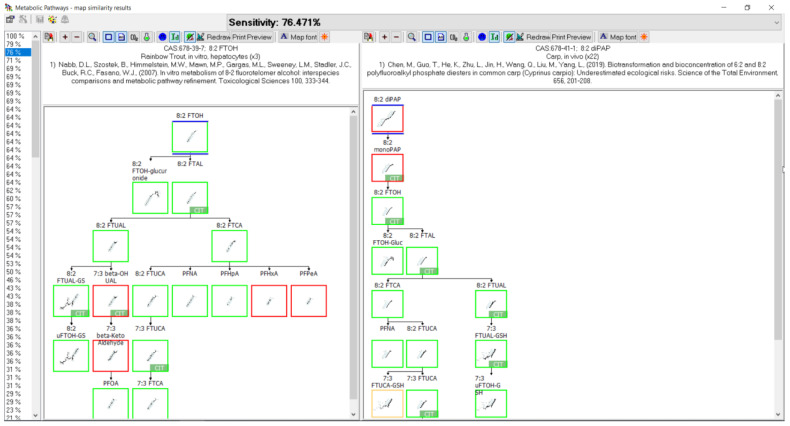
Metabolic map comparison of (**left**) 8:2 FTOH in rainbow trout hepatocytes and (**right**) 8:2 diPAP in vivo carp. Metabolites common to both species are indicated by green boxes. Differences in metabolites are indicated by red boxes. Tan boxes indicate phase II conjugates. Sensitivity, in this case 76.471%, is an assessment of the map comparison whereby the number of common metabolites is divided by the total number of metabolites in the fish map. Metabolites labeled with CIT are presumed intermediates in the path but were not identified in the study.

When reviewing the comparative analysis for the 10 PFAS examined here, the metabolic pathways appeared to be relatively well conserved across species and systems. A prime example is shown in Figure 16 whereby a direct comparison of rainbow trout hepatocyte and chicken in vivo maps for the biotransformation of 8:2 FTOH were compared. The structures highlighted with green boxes were common to both species, with those in red not conserved, and the structures in tan boxes identified as conjugates. Of the four structures identified in red for the rainbow trout, three were labeled as “CIT” and were presumed by the authors in their presentation of the metabolic pathway. This means they were not actually identified in the study but were presumed intermediates by the original authors when presenting their metabolic pathways. When the presumed intermediates are not considered, the comparison of maps between species improves and is actually very accurate. This observation is further supported by comparison of the 8:2 FTOH map for rainbow trout hepatocytes with the maps for rat (Figure 17) and pig (Figure 18). A direct comparison of the 8:2 FTOH map for rainbow trout hepatocytes was compared to the map for another fish species, carp in vivo with 8:2 diPAP (Figure 19). The 8:2 diPAP in carp forms 8:2 FTOH as a primary product and the resulting metabolic pathway displays a high level of congruence to the map observed in trout. There were differences in metabolite identification, but those were likely due to the diversity of experimental design and methods of analytical detection. When comparing metabolic maps across species and systems, several considerations arise dependent on the sample matrix analyzed and the ability to detect metabolites. As illustrated in the North Sea predators and canine examples above, there can and do exist true species differences but those seem to be more of a rare occurrence. The evidence gathered to this point would suggest the best estimate for the prediction of metabolites for PFAS lacking a fish biotransformation study would be to use the composite map formed by a combination of all other contributing maps.

## Data Availability

The compiled MetaPath database is available for download as Supplementary Material.

## Supplementary Materials

The following supporting information can be downloaded at: https://www.mdpi.com/article/10.3390/toxics11010074/s1, Database S1: PFAS Open Literature Database_2022_v.3.3.1; Database S2: PFAS Open Literature Database 2022 v.3.4.0.
